# Resveratrol Ameliorates Hyperuricemia in Association with Remodeling of the Gut Microbiota–Metabolism Axis: An Integrated 16S rDNA Sequencing and Fecal Metabolomics Study

**DOI:** 10.3390/foods15203686

**Published:** 2026-10-16

**Authors:** Yan Wang, Jing Xie, Zuoming Cao, Xiaoyu Gao, Zuoting Yang, Yang Tian, Jun Sheng, Lei Peng

**Affiliations:** 1 Key Laboratory of Development and Utilization of Food and Medicinal Resources, Ministry of Education, Yunnan Agricultural University, Kunming 650201, China; 2018039@ynau.edu.cn (Y.W.); jingxie0624@163.com (J.X.); 2018014@ynau.edu.cn (X.G.); yzt94448@163.com (Z.Y.); tianyang1208@163.com (Y.T.); 2 Engineering Research Center of Development and Utilization of Food and Drug Homologous Resources, Ministry of Education, Yunnan Agricultural University, Kunming 650201, China; 15298198413@163.com; 3 Yunnan Key Laboratory of Precision Nutrition and Personalized Food Manufacturing, Yunnan Agricultural University, Kunming 650201, China; 4 College of Food Science and Technology, Yunnan Agricultural University, Kunming 650201, China

**Keywords:** resveratrol, hyperuricemia, gut microbiota, 16S rDNA sequencing, untargeted metabolomics, multi-omics integration

## Abstract

Hyperuricemia (HUA) is a chronic metabolic disorder whose prevalence continues to rise, whereas available urate-lowering drugs are limited by safety and tolerability concerns. Resveratrol (Res) exhibits urate-lowering activity, but its oral bioavailability is below 1%, prompting investigation of its potential interactions with the gut microbiota. Here, hyperuricemic mice induced by potassium oxonate plus high-fructose intake were treated with Res (20, 40, or 80 mg/kg) or benzbromarone (20 mg/kg), and 16S rDNA sequencing, PICRUSt2 functional prediction, untargeted fecal metabolomics, and multi-omics network analysis were integrated to characterize the underlying mechanisms. Res (80 mg/kg) significantly lowered serum uric acid, inhibited xanthine oxidase and adenosine deaminase activities, improved renal injury, suppressed pro-inflammatory cytokines, and up-regulated colonic barrier genes with reduced serum lipopolysaccharide. Res normalized inflated microbial richness, restored *Akkermansia* abundance, and PICRUSt2 prediction indicated down-regulation of the microbial purine-metabolism gene families *XOD/XDH* and *UOX* by 29.4% and 85.9%, respectively. Metabolomics identified 41 shared differential metabolites including eicosanoid lipid mediators and glycerophospholipids. Multi-omics exploratory network analysis suggested *Akkermansia* as a potential hub node associated with microbial remodeling, eicosanoid modulation, and host urate phenotypes, which may represent a “urate-production suppression plus microbiota–metabolism axis regulation” mode of action, providing preclinical evidence for Res as a candidate dietary intervention against HUA.

## 1. Introduction

Hyperuricemia (HUA) is a chronic metabolic disorder arising from disturbed purine metabolism or impaired uric acid excretion and is characterized by abnormally elevated serum uric acid (SUA). Driven by the increasing consumption of purine-rich diets and sugar-sweetened beverages, the prevalence of HUA continues to rise, exceeding 14% among Chinese adults and increasingly affecting younger populations [1]. Worldwide, high SUA affects roughly one in five people and shows an upward trend, and HUA has been recognized as an independent risk factor for metabolic syndrome, chronic kidney disease, and cardiovascular disease [2,3]. Clinically, the characteristic osteoarticular pain of gout typically precedes overt renal injury, as hyperuricemia first manifests as articular urate crystal deposition, while renal involvement often develops insidiously over years [4,5]. Early-stage hyperuricemic nephropathy is largely reversible with adequate urate-lowering therapy and dietary intervention; however, sustained urate burden may progress to chronic tubulointerstitial fibrosis and irreversible glomerular damage [4,6]. Thus, timely modulation of serum uric acid represents a critical window to prevent irreversible renal injury and improve long-term prognosis in HUA patients. A sustained uric acid burden activates the hepatic NLR family pyrin domain containing 3 (NLRP3) inflammasome [7] and promotes renal urate deposition with tubulointerstitial injury [8], eventually progressing to gout and multi-organ damage. Elucidating the pathogenesis of HUA and identifying safe, effective intervention strategies therefore carry considerable public health significance [3].

As the end product of purine metabolism, uric acid homeostasis depends on the dynamic balance between hepatic production and renal and intestinal elimination [9]. Hepatic xanthine oxidoreductase (XOR) catalyzes the oxidation of hypoxanthine to xanthine and then to uric acid, which is the rate-limiting step of urate production [10]; uric acid excretion is coordinated by renal urate transporters such as URAT1, GLUT9, and OAT10 [11,12], among which ABCG2 serves as an efflux pump at both the intestinal and renal barriers, and its dysfunction has emerged as an important cause of HUA [13,14,15]. Current clinical therapies, mainly allopurinol, febuxostat, and benzbromarone, effectively lower SUA but are constrained by hypersensitivity reactions, cardiovascular safety concerns, hepatotoxicity, and rebound after withdrawal [16,17,18,19]. Mining safe and sustainable urate-regulating factors from dietary ingredients has consequently become an important direction for nutritional intervention in HUA [16,17].

Accumulating evidence indicates that the gut microbiota has a significant role in regulating uric acid homeostasis through purine degradation, urate efflux, short-chain fatty acid (SCFA) production, and regulation of the intestinal barrier [20,21]; certain *Lactobacillus* strains can directly degrade purine precursors [22]. Gut microbial composition clearly distinguishes gout patients from healthy individuals, with protective genera such as *Akkermansia* and *Faecalibacterium* being depleted [23]. Cohort studies have shown that baseline microbial features can predict the trajectory of SUA changes [24], and urate-lowering drugs themselves reshape the microbiota [25]. In animal experiments, oral administration of *Akkermansia muciniphila* alone significantly reduces SUA, inhibits xanthine oxidase (XOD), and repairs the intestinal barrier [26]. Subsequent studies confirmed that both pasteurized and heat-inactivated *A. muciniphila* alleviate HUA by suppressing the Toll-like receptor 4 (TLR4)/NF-κB/NLRP3 inflammatory cascade, modulating ABCG2/GLUT9 urate transporters, and raising SCFA levels [27], with consistent effects even in an avian model [28], which suggests a robust, cross-model urate-lowering effect. In addition, the microbial metabolite butyrate was reported to reverse aging-associated gout susceptibility [29], further supporting the feasibility of the “microbiota–metabolism” axis as an intervention target. Most of these studies, however, have focused on direct supplementation with single strains (e.g., *Akkermansia* and *Lactobacillus*) or on phenotypic associations with single metabolites (e.g., SCFAs); systematic multi-omics evidence linking microbial remodeling, metabolic output, and improved host urate phenotypes remains scarce [20].

Resveratrol (Res), a polyphenolic active component of grapes, Polygonum cuspidatum, and other plants [30], has an oral bioavailability below 1%, with the vast majority retained in the gut, where it interacts extensively with the microbiota [31]; its in vivo effects may therefore depend heavily on microbial mediation [32,33]. Earlier studies demonstrated that Res lowers SUA in HUA model animals [34,35], but mechanistic work has centered on direct hepatic XOD inhibition and renal transcriptional regulation, leaving it unclear whether the gut microbiota mediates the urate-lowering effect of Res. Combining metagenomics, 16S rRNA sequencing, and fecal microbiota transplantation, Zhou et al. (2024) showed that Res alleviates high-fat-diet-induced HUA by remodeling the gut microbiota, enhancing intestinal uric acid degradation, and up-regulating microbial purine-metabolism functions [36], establishing the necessity of the microbiota for the effect of Res. That study, however, did not incorporate metabolomics to resolve the overall metabolic output of the microbiota, making it difficult to clarify through which metabolites the microbiota connects to host uric acid homeostasis, and it did not benchmark Res against classic urate-lowering drugs.

Integrated 16S rDNA sequencing and fecal metabolomics approaches have been successfully employed to characterize gut microbial and metabolic alterations in various disease models, including inflammatory bowel disease [37], and glucose and lipid metabolism disturbances [38], demonstrating the robustness of this dual-omics strategy for elucidating microbiota–metabolism interactions. We therefore hypothesized that Res lowers uric acid not merely by directly inhibiting hepatic XOD, but by remodeling gut microbial composition, suppressing hyperactivated microbial purine metabolism, and modulating eicosanoid and other inflammatory lipid mediators, thereby improving the intestinal barrier, alleviating systemic inflammation, and ultimately reducing SUA. To test this hypothesis, we used a mouse model of HUA induced by potassium oxonate combined with high-fructose intake, treated the animals with low, medium, and high doses of Res, and used benzbromarone as a positive control. By integrating 16S rDNA sequencing, PICRUSt2 functional prediction, untargeted fecal metabolomics, and multi-omics integration analysis, we systematically characterized the microbial and metabolic mechanisms underlying the effect of Res on HUA, providing a scientific basis for the development of Res-based functional foods and for the dietary management of HUA.

## 2. Materials and Methods

### 2.1. Chemicals and Reagents

Resveratrol (Res, CAS 501-36-0), potassium oxonate (PO, CAS 2207-75-2), and benzbromarone (BEN, CAS 3562-84-3) were purchased from Shanghai Yuanye Bio-Technology Co., Ltd. (Shanghai, China). D-Fructose was obtained from Amresco (Solon, OH, USA), and sodium carboxymethyl cellulose (CMC-Na) from Sinopharm Chemical Reagent Co., Ltd. (Shanghai, China). Assay kits for uric acid (UA) and creatinine in serum and urine, serum xanthine oxidase (XOD) and adenosine deaminase (ADA) activities, blood urea nitrogen (BUN), tumor necrosis factor-α (TNF-α), interleukin-1β (IL-1β), interleukin-6 (IL-6), and lipopolysaccharide (LPS) were purchased from Nanjing Jiancheng Bioengineering Institute (Nanjing, China). Reagents for total RNA extraction, reverse transcription, and qRT-PCR were purchased from TaKaRa (Kusatsu, Japan). All other reagents were of analytical grade.

### 2.2. Instruments

The main instruments were as follows: individually ventilated cages (S8; Suzhou Suhang Technology Co., Ltd., Suzhou, China); a LightCycler 480 II real-time PCR system (Roche, Mannheim, Germany); an Epoch 2 microplate reader (BioTek, Winooski, VT, USA); an Olympus CX43 microscope (Olympus, Tokyo, Japan); a Leica RM2235 paraffin microtome (Leica, Wetzlar, Germany); a BPG-9156A electrothermal drying oven (Shanghai Yiheng, Shanghai, China); a Micro Pure UV water purification system; an ultra-high performance liquid chromatography (UHPLC)–Orbitrap Exploris 240 high-resolution mass spectrometer (Thermo Fisher Scientific, Waltham, MA, USA); and an Illumina MiSeq high-throughput sequencing platform (Shanghai Meiji Biomedical Technology Co., Ltd., Shanghai, China).

### 2.3. Animals and Experimental Design

Thirty-six male C57BL/6J mice (SPF grade, 6 weeks old, 20–25 g) were obtained from Liaoning Changsheng Biotechnology Co., Ltd. (Benxi, China) and kept in individually ventilated cages with corncob bedding (3 mice per cage) at 22–25 °C and 45–55% relative humidity on a 12 h light/dark cycle (lights on 07:00–19:00), with food and water available ad libitum; no additional environmental enrichment items (e.g., nesting material, shelters, or toys) were provided. After 7 days of acclimation, the animals were randomly allocated at the cage level to six groups (*n* = 6 per group, two cages per group, each cage housing animals from a single group only) using a random number generator (Microsoft Excel 2019), with body weight balanced across groups (baseline body weight did not differ among groups, one-way ANOVA, *p* > 0.05): a blank control group (CON), a model group (HUA), a benzbromarone positive-control group (BEN, 20 mg/kg), and three resveratrol dose groups (Res 20, Res 40, and Res 80 mg/kg). Except for the CON group, all mice received intraperitoneal injections of potassium oxonate (PO, 250 mg/kg, dissolved in saline containing Tween-80, pH 7.6) and drank freely available 5% fructose water for four weeks to induce hyperuricemia; CON mice received equal volumes of saline and drank distilled water. Starting from the first day of modeling, the BEN and Res groups were given the corresponding drug suspensions (approximately 200 μL) by gavage every morning at 10:00 for four consecutive weeks, whereas the CON and HUA groups received an equal volume of 0.5% CMC-Na. Body weight and food intake were monitored once weekly. The animal protocol was reviewed and approved by the Ethics Committee for Life Sciences of Yunnan Agricultural University (approval No. YNAU-202308008). No animals or data points were excluded from the analysis.

Cages of different groups were interspersed on the same rack and rotated weekly to minimize rack-position bias. Because 5% fructose water (or distilled water for CON) was supplied from one bottle per cage, all cage-mates received identical fluid exposure, and all bottles were refilled simultaneously; for the 16S rDNA sequencing and metabolomics analyses, cecal contents and fecal samples, respectively, were collected from all six mice per group (both cages), so that no single cage dominated the omics dataset. For outcome assessment, serum biochemical assays, histopathological assessment, and sequencing-based analyses were performed using coded sample identities by investigators blinded to group allocation, and statistical analysis was performed on a coded dataset with group decoding completed after analysis. Mice were inspected daily by trained animal-care staff, and predefined humane endpoints (>20% loss of baseline body weight, persistent hunched posture with ruffled fur, marked lethargy, inability to access food or water, or severe diarrhea) were not reached by any animal. At sacrifice, after the 12 h fast, mice were euthanized by gradual-fill CO_2_ inhalation (approximately 30–70% of chamber volume per minute) followed by cervical dislocation, and death was confirmed by the absence of pedal and corneal reflexes. The study followed the 3Rs: xanthine oxidase inhibition assays preceded the animal study (Replacement); the number of animals was minimized, and cecal contents and feces from the same animals were analyzed by 16S rDNA sequencing and untargeted metabolomics, respectively (Reduction); and humane endpoints, daily monitoring and gradual-fill CO_2_ euthanasia minimized pain and distress (Refinement).

The sample size (*n* = 6 per group) was determined based on prior studies employing similar HUA mouse models and multi-omics approaches, which have demonstrated sufficient statistical power with this group size [39]. Mice were randomly assigned to cages (3 mice per cage, 2 cages per group) and treatment groups to minimize systematic bias. We acknowledge that cage effects were not included as a covariate in the statistical analysis; however, the randomization of mice across cages and the use of multiple cages per group were designed to distribute potential cage-related variability evenly across treatment groups. Future studies with larger sample sizes should formally evaluate and account for cage effects in the statistical model.

### 2.4. Sample Collection and Biochemical Measurements

The primary outcome measure was serum uric acid (SUA). After the final administration, the mice were fasted for 12 h with free access to water. The 12 h fasting duration was selected to standardize metabolic baseline conditions while avoiding excessive physiological stress associated with prolonged fasting (>16 h), which can induce hepatic glycogen depletion, altered bile acid metabolism, and compensatory lipogenesis that confound serum uric acid and metabolomic measurements. A 12 h fasting window represents a standard compromise between achieving consistent post-absorptive metabolic states and minimizing stress-related perturbations of gut microbiota composition and host metabolism in murine models [40,41]. Blood, urine, kidney, colon, and cecal contents were collected. Uric acid and creatinine in serum and urine, serum XOD and ADA activities, and serum BUN were measured according to the manufacturers’ instructions.

### 2.5. Histopathological Examination

Kidney and colon tissues were fixed in 4% paraformaldehyde, routinely embedded in paraffin, and sectioned at 5 μm. Sections were stained with hematoxylin and eosin (H&E). Consistent with common practice in hyperuricemia animal studies, histopathological changes were evaluated qualitatively rather than by a semiquantitative scoring system: for kidney sections, glomerular architecture, tubular epithelial integrity, vacuolar degeneration, and interstitial inflammatory infiltration were examined; for colon sections, mucosal architecture, epithelial integrity, goblet cell abundance, and inflammatory cell infiltration were examined. All sections were assessed independently by an experienced pathologist blinded to group allocation, using coded slides. Representative images were captured under a light microscope to illustrate the histological features of each group.

### 2.6. Serum Inflammatory Cytokines and Colonic Barrier Gene Expression

Serum TNF-α, IL-6, IL-1β, and LPS were measured by ELISA.

For qRT-PCR analysis, total RNA was extracted from colon tissue using the TaKaRa MiniBEST Universal RNA Extraction Kit (TaKaRa, Japan) according to the manufacturer’s instructions. RNA concentration and purity were assessed using a micro-spectrophotometer (A260/A280 ratio between 1.8–2.0). One microgram of total RNA was reverse-transcribed to cDNA using the PrimeScript™ RT reagent Kit with gDNA Eraser (Perfect Real Time) (TaKaRa, Japan) to remove genomic DNA. qRT-PCR was performed using TB Green^®^ Premix Ex Taq™ II (Tli RNaseH Plus) (TaKaRa, Japan) in a 20 μL reaction volume on a LightCycler^®^ 480 II Real-Time PCR System (Roche, Mannheim, Switzerland). The amplification program consisted of initial denaturation at 95 °C for 30 s, followed by 40 cycles of denaturation at 95 °C for 10 s, and annealing/extension at 60 °C for 30 s. A melting curve analysis was performed after each run to confirm primer specificity. Each sample was analyzed in duplicate with *GAPDH* as the internal reference, and relative expression was calculated by the 2^−ΔΔCt method. Primer sequences are listed in Table 1.

### 2.7. Gut Microbiota 16S rDNA Sequencing

Cecal contents were collected, snap-frozen in liquid nitrogen, and stored at −80 °C. 16S rDNA high-throughput sequencing was performed by Shanghai Meiji Biomedical Technology Co., Ltd. Genomic DNA was extracted, and the V3–V4 hypervariable region was amplified with primers 338F (5′-ACTCCTACGGGAGGCAGCAG-3′) and 806R (5′-GGACTACHVGGGTWTCTAAT-3′), followed by paired-end 300 bp sequencing on the Illumina MiSeq platform. After quality control and read merging, amplicon sequence variants (ASVs) were clustered and taxonomically annotated against the SILVA 138 database. Samples were rarefied to the minimum sequencing depth before α-diversity (Ace, Chao, Shannon, Simpson, and Sobs indices), β-diversity (PCoA and NMDS), and LEfSe analyses. The raw sequencing data were deposited in the NCBI Sequence Read Archive under accession number PRJNA1531718. Differential taxa were identified by LEfSe with an LDA score > 2.0, and *p* values were adjusted by the Benjamini–Hochberg false discovery rate (FDR) procedure (q < 0.05).

### 2.8. Functional Prediction of the Microbiota

Based on the 16S rDNA sequencing data, microbial functions were predicted with Phylogenetic Investigation of Communities by Reconstruction of Unobserved States 2 (PICRUSt2, v2.6.2) against the KEGG database, with particular attention to gene family abundances in purine metabolism (ko00230) and pyrimidine metabolism (ko00240). The analyses were performed on the Majorbio cloud platform (cloud.majorbio.com, accessed on 7 September 2026).

### 2.9. Fecal Untargeted Metabolomics Analysis

Sample Preparation and Quality Control: Fecal samples (100 mg) were placed in 2 mL centrifuge tubes containing a 6 mm grinding bead and mixed with 800 μL of extraction solvent (methanol:water = 4:1, *v*/*v*) containing the internal standard L-2-chlorophenylalanine (0.02 mg/mL). Samples were ground in a frozen tissue grinder for 6 min (−10 °C, 50 Hz), followed by low-temperature ultrasonic extraction for 30 min (5 °C, 40 kHz). After extraction, samples were incubated at −20 °C for 30 min and centrifuged at 13,000× *g* for 15 min at 4 °C. The supernatant was transferred to autosampler vials for UHPLC-Orbitrap analysis. For quality control, pooled QC samples were prepared by mixing equal aliquots from each experimental sample and inserted every 10 samples to monitor instrument stability.

UHPLC-Orbitrap MS Analysis: Chromatographic separation was performed on an HSS T3 column (100 mm × 2.1 mm i.d., 1.8 μm; Waters, Milford, MA, USA) with mobile phase A (water/acetonitrile, 95:5, *v*/*v*, containing 0.1% formic acid) and mobile phase B (acetonitrile/isopropanol/water, 47.5:47.5:5, *v*/*v*/*v*, containing 0.1% formic acid) under gradient elution at a flow rate of 0.40 mL/min, column temperature of 40 °C, and injection volume of 5 μL. Mass spectrometry was conducted with electrospray ionization (ESI) in both positive and negative modes (spray voltage: 3500 V positive, −3000 V negative; sheath gas: 50 arb; auxiliary gas: 13 arb; capillary temperature: 450 °C). Full MS/dd-MS2 acquisition was used with full scan resolution 60,000 (*m*/*z* 200) and MS/MS resolution 15,000 (*m*/*z* 200). Stepped normalized collision energy (NCE) was set at 20%, 40%, and 60%.

Data Preprocessing and Analysis: Raw data were processed using Progenesis QI software (Waters Corporation, Milford, MA, USA) for baseline filtering, peak detection, integration, retention time correction, and alignment. Metabolites were identified by matching MS and MS/MS spectra against HMDB (http://www.hmdb.ca/, accessed on 7 September 2026), Metlin (https://metlin.scripps.edu/, accessed on 7 September 2026), and the Majorbio in-house database (Level 2 confidence). Data preprocessing was performed on the Majorbio Cloud Platform: (1) missing values were filtered using the 80% rule; (2) remaining missing values were imputed using the minimum value method; (3) peak intensities were normalized by sum normalization; (4) features with relative standard deviation (RSD) >30% in QC samples were excluded; and (5) data were log10-transformed. Group differences in metabolic profiles were evaluated by principal component analysis (PCA) and orthogonal partial least squares discriminant analysis (OPLS-DA), with model reliability verified by 200 permutation tests. Differential metabolites were screened at VIP > 1.0 with FDR-adjusted *p* < 0.05 (Benjamini–Hochberg procedure) and were annotated and subjected to metabolic pathway enrichment analysis based on the HMDB and KEGG databases.

### 2.10. Integration Analysis of Multi-Omics Data

Only the CON, HUA, and RES groups (18 samples in total) were included in the integration analysis; the BEN group was not entered into the network because of the experimental design. Integrating the gut microbiota data from 16S rDNA sequencing with the fecal metabolomics data, we constructed a “microbiota–metabolite–host phenotype” interaction network. Spearman rank correlation coefficients [42] were calculated from the relative abundances of differential taxa and the peak areas of differential metabolites; correlations with |r| ≥ 0.47 and Benjamini–Hochberg-adjusted *p* < 0.05 were retained to build three association matrices visualized as heatmaps: (1) differential genera versus differential metabolites; (2) differential genera versus host biochemical indices; and (3) core differential metabolites versus host biochemical indices. The differential microbial taxa, differential metabolites, and host phenotypic data were then integrated to construct a three-layer “microbiota–metabolite–host” network. Community detection was performed with the greedy modularity maximization algorithm to identify functional modules. Node shapes indicate the omics layer (circle, microbiota; square, metabolite; diamond, host phenotype), and node colors indicate module membership. Network visualization and module analysis were performed in Cytoscape 3.10.4.

### 2.11. Statistical Analysis

All data are expressed as means ± standard deviations (SDs). Statistical analysis and graphing were performed with GraphPad Prism 9.0. Comparisons among multiple groups were made by one-way analysis of variance (ANOVA) followed by Tukey’s post hoc test. LEfSe analysis followed the standard protocol described by Segata et al. [43]. For 16S rDNA sequencing, untargeted metabolomics, and omics correlation analyses, *p* values were adjusted by the Benjamini–Hochberg false discovery rate (FDR) procedure (q < 0.05), together with the effect-size criteria described in Section 2.10. Differences were considered significant at *p* (or adjusted q) < 0.05 (two-tailed).

## 3. Results

### 3.1. Effects of Res on SUA and Serum XOD and ADA Activities in HUA Mice

**Figure 1:**
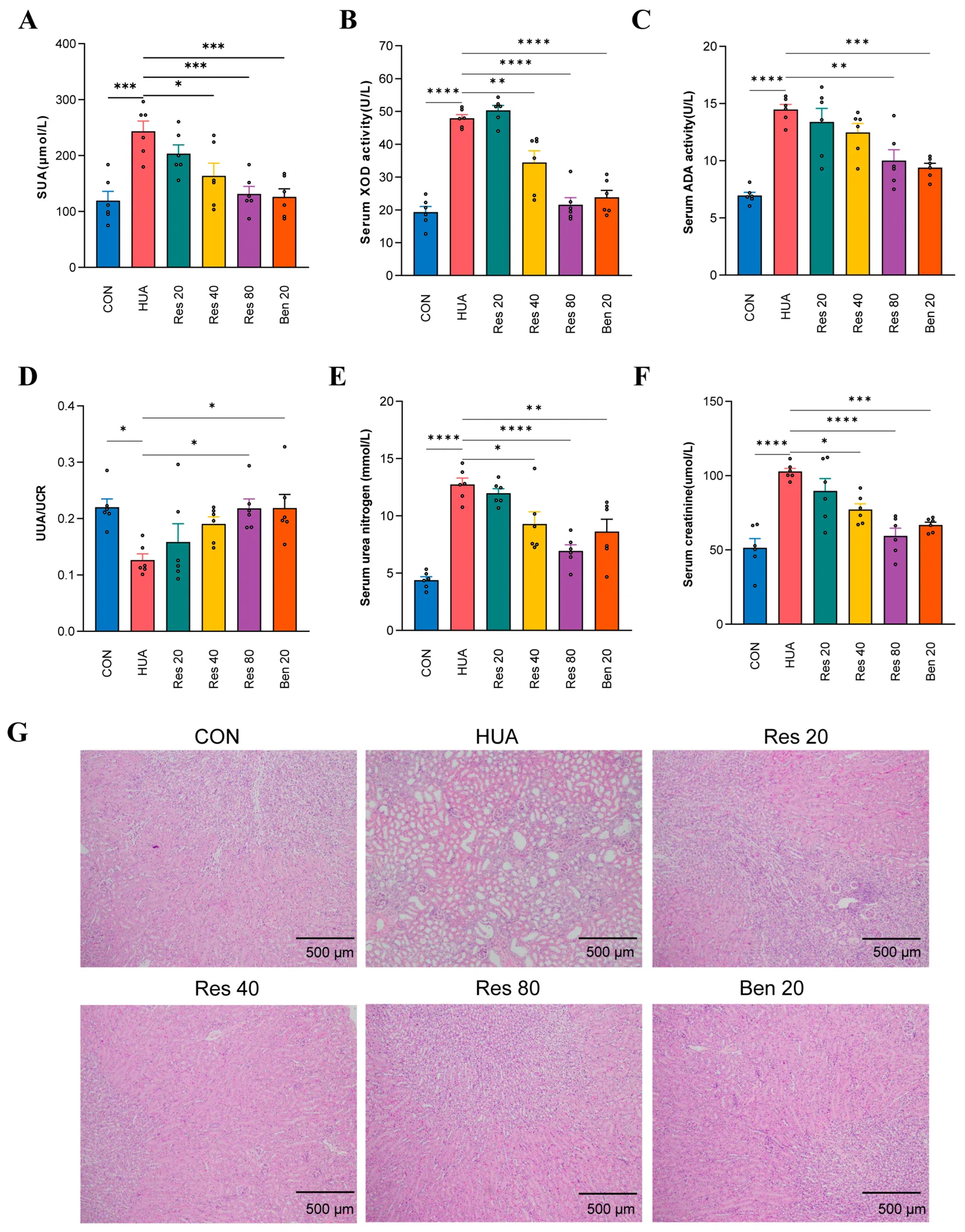
Effects of resveratrol on serum uric acid, purine-metabolizing enzyme activities, uric acid excretion, renal function, and renal pathology in hyperuricemic mice. (**A**) Serum uric acid (SUA); (**B**) serum xanthine oxidase (XOD) activity; (**C**) serum adenosine deaminase (ADA) activity; (**D**) urinary uric acid/urinary creatinine ratio (UUA/UCr); (**E**) serum blood urea nitrogen (BUN); (**F**) serum creatinine (CRE); (**G**) representative hematoxylin–eosin (H&E) staining of kidney tissue (×100; scale bar = 500 μm). Data are means ± SD (*n* = 6). * *p* < 0.05, ** *p* < 0.01, *** *p* < 0.001, **** *p* < 0.0001 vs. the HUA group.

The SUA level of the HUA group was significantly higher than that of the CON group (*p* < 0.0001), confirming successful modeling (Figure 1A–C). After Res intervention, SUA decreased in a dose-dependent manner: the reduction was not significant at 20 mg/kg, became significant at 40 mg/kg (*p* < 0.05), and was further enhanced at 80 mg/kg (*p* < 0.001), approaching the levels of the CON and BEN groups. In line with the changes in SUA, serum XOD and ADA activities were significantly elevated in the HUA group compared with the CON group (both *p* < 0.0001). Res at 40 and 80 mg/kg significantly inhibited XOD activity (*p* < 0.01 and *p* < 0.0001, respectively), whereas 20 mg/kg had no significant effect; ADA activity was unchanged at 20 and 40 mg/kg but was significantly reduced at 80 mg/kg (*p* < 0.01).

### 3.2. Effects of Res on Uric Acid Excretion in HUA Mice

Compared with the CON group, the urinary uric acid/urinary creatinine ratio (UUA/UCr) was significantly reduced in the HUA group (*p* < 0.05), indicating disturbed renal tubular urate handling under hyperuricemic conditions, with an abnormally low proportion of filtered uric acid being excreted. Res at 20 and 40 mg/kg had no significant effect on the UUA/UCr ratio. Res at 80 mg/kg significantly increased the ratio (*p* < 0.05) to near-normal levels, indicating restored urate excretion in the renal tubules under hyperuricemic conditions, with a normalized proportion of uric acid excreted per unit of glomerular filtration. The positive control benzbromarone (BEN, 20 mg/kg) also significantly increased the UUA/UCr ratio (*p* < 0.05), consistent with its uricosuric effect and attenuation of tubulointerstitial injury. These results indicate that both Res and benzbromarone ameliorate the disturbance of renal urate handling in HUA mice, albeit through distinct mechanisms.

### 3.3. Effects of Res on Renal Function and Renal Histopathology

Serum creatinine (CRE) and BUN were markedly elevated in the HUA group compared with the CON group (both *p* < 0.0001), indicating impaired renal function (Figure 1E,F). Res at 20 mg/kg did not significantly improve these indices, whereas 40 mg/kg significantly reduced both CRE and BUN (both *p* < 0.05); the effect was stronger at 80 mg/kg (CRE *p* < 0.0001; BUN *p* < 0.0001), approaching normal values. Histopathological examination (Figure 1G) showed intact glomerular and tubular structures in the CON group. In contrast, the HUA group exhibited disorganized glomeruli, vacuolar degeneration and necrotic shedding of tubular epithelial cells, and massive inflammatory cell infiltration in the interstitium. These lesions were attenuated by Res in a dose-dependent manner, with the Res 80 group approaching the CON morphology; the BEN group also showed marked improvement.

### 3.4. Effects of Res on Serum Inflammatory Cytokines and the Colonic Mucosal Barrier

**Figure 2:**
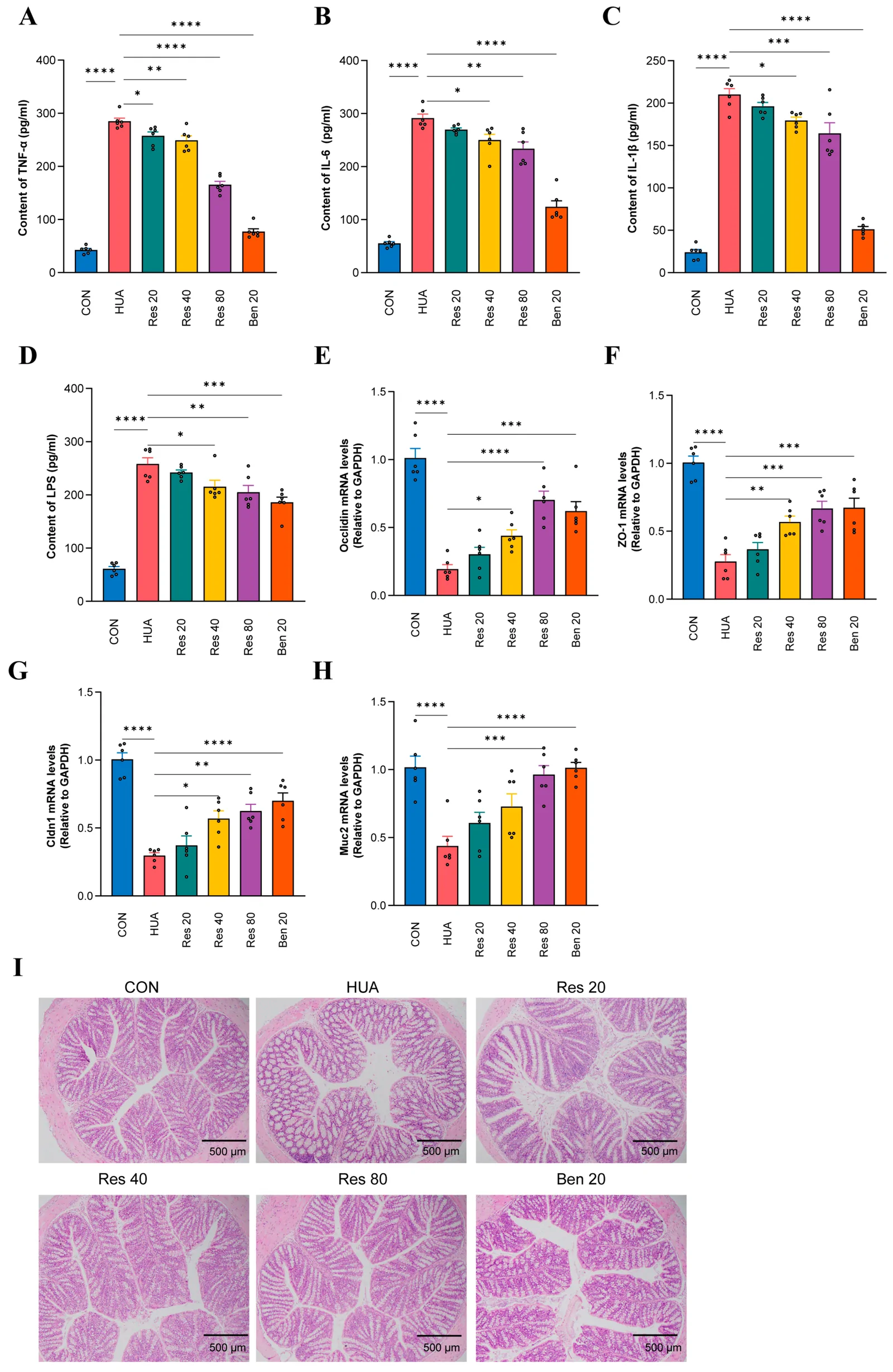
Effects of resveratrol on serum inflammatory cytokines, intestinal barrier function, and colonic pathology in hyperuricemic mice. (**A**) Serum TNF-α; (**B**) serum IL-6; (**C**) serum IL-1β; (**D**) serum LPS; (**E**–**H**) relative mRNA expression of colonic *Occludin* (**E**), *ZO-1* (**F**), *Claudin-1* (**G**), and *Muc2* (**H**); (**I**) representative H&E staining of colon tissue (×100; scale bar = 500 μm). Data are means ± SD (*n* = 6). * *p* < 0.05, ** *p* < 0.01, *** *p* < 0.001, **** *p* < 0.0001.

Serum TNF-α, IL-6, and IL-1β were all significantly elevated in the HUA group relative to the CON group (all *p* < 0.0001) (Figure 2A–C). The inhibition of TNF-α by Res was already evident at the lowest dose (Res 20, *p* < 0.05), was enhanced at 40 mg/kg (*p* < 0.001), and was strongest at 80 mg/kg (*p* < 0.0001). IL-6 did not change significantly in the Res 20 group but was significantly reduced at 40 mg/kg (*p* < 0.05) and 80 mg/kg (*p* < 0.01); IL-1β followed the same pattern (Res 40, *p* < 0.05; Res 80, *p* < 0.001). At 80 mg/kg, TNF-α, IL-1β, and IL-6 fell by approximately 42%, 23%, and 20% relative to the HUA group, respectively. The three cytokines were also significantly reduced in the BEN group (all *p* < 0.0001), approaching CON levels.

Regarding the colonic mucosal barrier, qRT-PCR showed that colonic *Occludin*, *ZO-1*, *Claudin-1*, and *Muc2* mRNA levels were all significantly down-regulated in the HUA group (all *p* < 0.0001) (Figure 2E–H). Meanwhile, serum LPS (Figure 2D) rose from approximately 60 pg/mL in the CON group to approximately 255 pg/mL in the HUA group (*p* < 0.0001, about 4.3-fold). Res at 20 mg/kg did not significantly restore these indices. Res at 40 mg/kg significantly restored *Occludin* (*p* < 0.05), *ZO-1* (*p* < 0.01), and Claudin-1 (*p* < 0.05), without significantly affecting Muc2. Res at 80 mg/kg significantly up-regulated all four genes (*Occludin p* < 0.0001; *ZO-1 p* < 0.001; *Claudin-1 p* < 0.01; *Muc2 p* < 0.001) and significantly lowered serum LPS to approximately 200 pg/mL (*p* < 0.01), although not to CON levels. Of note, all four barrier genes were significantly restored in the BEN group (*p* < 0.001–0.0001), and LPS fell to approximately 185 pg/mL (*p* < 0.01), a reduction even greater than that in the Res 80 group, suggesting that lowering SUA per se indirectly improves intestinal barrier integrity. The multi-dimensional regulation of tight junction proteins by Res (especially at 40–80 mg/kg), however, points to a direct gut-protective effect beyond urate lowering. H&E staining of the colon showed epithelial disruption, damaged crypt architecture, and dense mucosal inflammatory infiltration in HUA mice, all of which were dose-dependently restored by Res (Figure 2I).

### 3.5. Remodeling of the Gut Microbial Community

**Figure 3:**
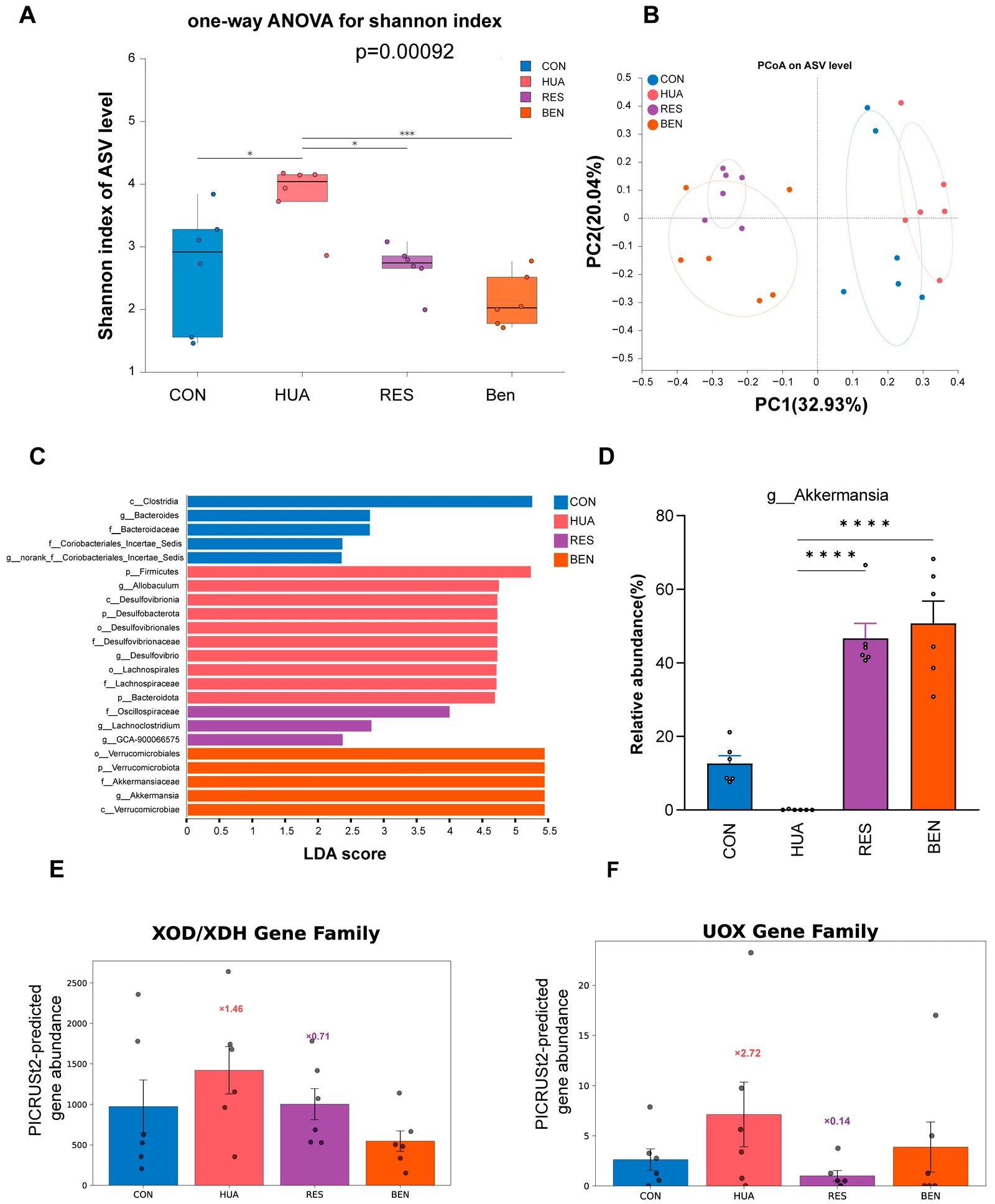
Resveratrol reshapes gut microbial diversity, community structure, and predicted purine-metabolism functions in hyperuricemic mice. (**A**) Box plot of the Shannon index (α-diversity); (**B**) PCoA based on Bray–Curtis distances (β-diversity); (**C**) LEfSe LDA score bar plot (LDA > 2.0); (**D**) intergroup comparison of the relative abundance of *Akkermansia*; (**E**) PICRUSt2-predicted abundance of the *XOD/XDH* gene family; (**F**) PICRUSt2-predicted abundance of the *UOX* gene family. Data are means ± SD (*n* = 6). * *p* < 0.05, *** *p* < 0.001, **** *p* < 0.0001.

Because the 80 mg/kg dose of Res showed the most pronounced urate-lowering, anti-inflammatory, and gut barrier-repairing effects, we focused on the highest dose for 16S rDNA sequencing and fecal metabolomics, comparing the CON, HUA, RES (80 mg/kg), and BEN (20 mg/kg) groups. α-Diversity analysis (Figure 3A) showed that the Shannon index was significantly higher in the HUA group than in the CON group (*p* < 0.05), a dysbiosis pattern of inflated richness with imbalanced structure. Res at 80 mg/kg significantly restored the Shannon index (*p* < 0.05 versus HUA), and all diversity indices returned to levels not significantly different from the CON group. BEN also significantly reversed these changes (*p* < 0.01), suggesting that urate lowering alone partially corrects microbial dysbiosis. PCoA of β-diversity (Figure 3B) clearly separated the CON and HUA groups, whereas the RES and BEN samples shifted away from HUA toward the CON cluster. LEfSe analysis (Figure 3C,D) identified Firmicutes- and Desulfobacterota-related lineages as enriched in the HUA group, whereas the RES group was characteristically enriched in *Akkermansia* of the phylum Verrucomicrobiota. At the genus level, *Akkermansia* was significantly depleted and *Desulfovibrio* enriched in the HUA group; Res at 80 mg/kg significantly restored *Akkermansia* and reduced *Desulfovibrio*. Although the BEN group was likewise enriched in *Akkermansia*, its microbial regulatory effect appeared largely confined to the Verrucomicrobiota lineage, whereas Res at 80 mg/kg modulated multiple phyla including Verrucomicrobiota, Desulfobacterota, and Firmicutes, suggesting a broader impact on the overall community structure.

### 3.6. PICRUSt2 Functional Prediction

PICRUSt2 functional prediction (Figure 3E,F) suggested alterations in the purine metabolism pathway. The predicted abundances of the microbial xanthine oxidoreductase/xanthine dehydrogenase (*XOD/XDH*) and urate oxidase (*UOX*) gene families were abnormally up-regulated in the HUA group, by 45.8% and 172.1%, respectively, suggesting hyperactivated microbial purine degradation under hyperuricemic conditions. Res at 80 mg/kg was predicted to pull these elevations back by 29.4% and 85.9%, respectively, suggesting potential restoration of microbial purine-metabolic homeostasis. It is important to note that these are predicted functional profiles derived from 16S rDNA sequences rather than direct measurements of gene expression or enzymatic activity.

### 3.7. Untargeted Fecal Metabolomics

**Figure 4:**
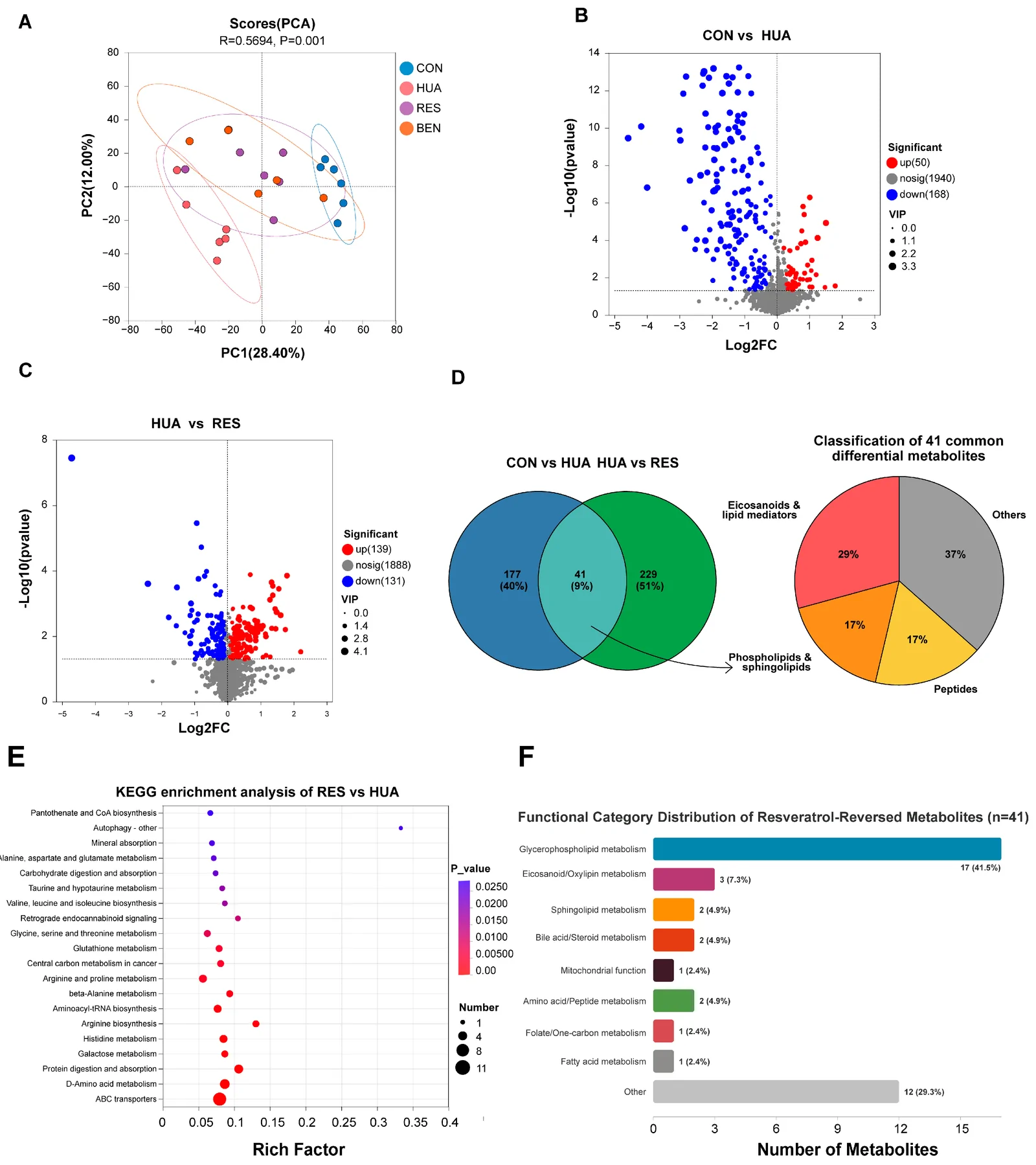
Resveratrol alters the fecal metabolic profile and differential metabolites in hyperuricemic mice. (**A**) PCA score plot of fecal metabolomes from the four groups; (**B**) volcano plot of differential metabolites for CON vs. HUA; (**C**) volcano plot of differential metabolites for HUA vs. RES; (**D**) Venn diagram of differential metabolites shared by CON vs. HUA and HUA vs. RES and functional classification pie chart of the 41 shared metabolites; (**E**) KEGG pathway enrichment bubble plot of differential metabolites for HUA vs. RES; (**F**) bar chart of the chemical-category distribution of the 41 shared metabolites. Differential metabolites were screened at VIP > 1.0 and FDR-adjusted *p* < 0.05.

Untargeted metabolomics of fecal samples from the CON, HUA, RES (80 mg/kg), and BEN (20 mg/kg) groups was performed by UHPLC–Orbitrap high-resolution mass spectrometry. The PCA score plot showed clear separation of the four metabolic profiles (Figure 4A), and PERMANOVA based on Bray–Curtis distances confirmed that the intergroup differences were statistically significant (*R*^2^ = 0.5694, *p* = 0.001). OPLS-DA models further distinguished the group profiles; both the CON vs. HUA and HUA vs. RES models achieved *R*^2^*Y* > 0.9 and *Q*^2^ > 0.8, and 200 permutation tests verified the absence of overfitting.

Differential metabolites were screened at VIP > 1.0 and FDR-adjusted *p* < 0.05: 218 were identified: 218 were identified for CON vs. HUA (50 up-regulated and 168 down-regulated in HUA; Figure 4B) and 270 for HUA vs. RES (139 up-regulated and 131 down-regulated; Figure 4C). Overlap analysis yielded 41 differential metabolites shared by the two comparisons (Figure 4D, left panel). Manual functional classification based on the HMDB database showed that eicosanoids and lipid mediators constituted the largest category (29.3%), followed by phospholipids and sphingolipids (17.1%) and peptides (17.1%) (Figure 4D, right panel).

KEGG-based pathway enrichment further indicated arachidonic acid metabolism, linoleic acid metabolism, and α-linolenic acid metabolism as the main perturbed pathways (Figure 4E). Classification by chemical nature (a dimension different from the HMDB functional classification in Figure 4D) showed that glycerophospholipids accounted for the highest proportion (41.5%), followed by eicosanoids/oxylipins (7.3%) (Figure 4F), pointing to eicosanoid and phospholipid disturbances as the core feature of the fecal metabolome in HUA.

### 3.8. Multi-Omics Integration Analysis

**Figure 5:**
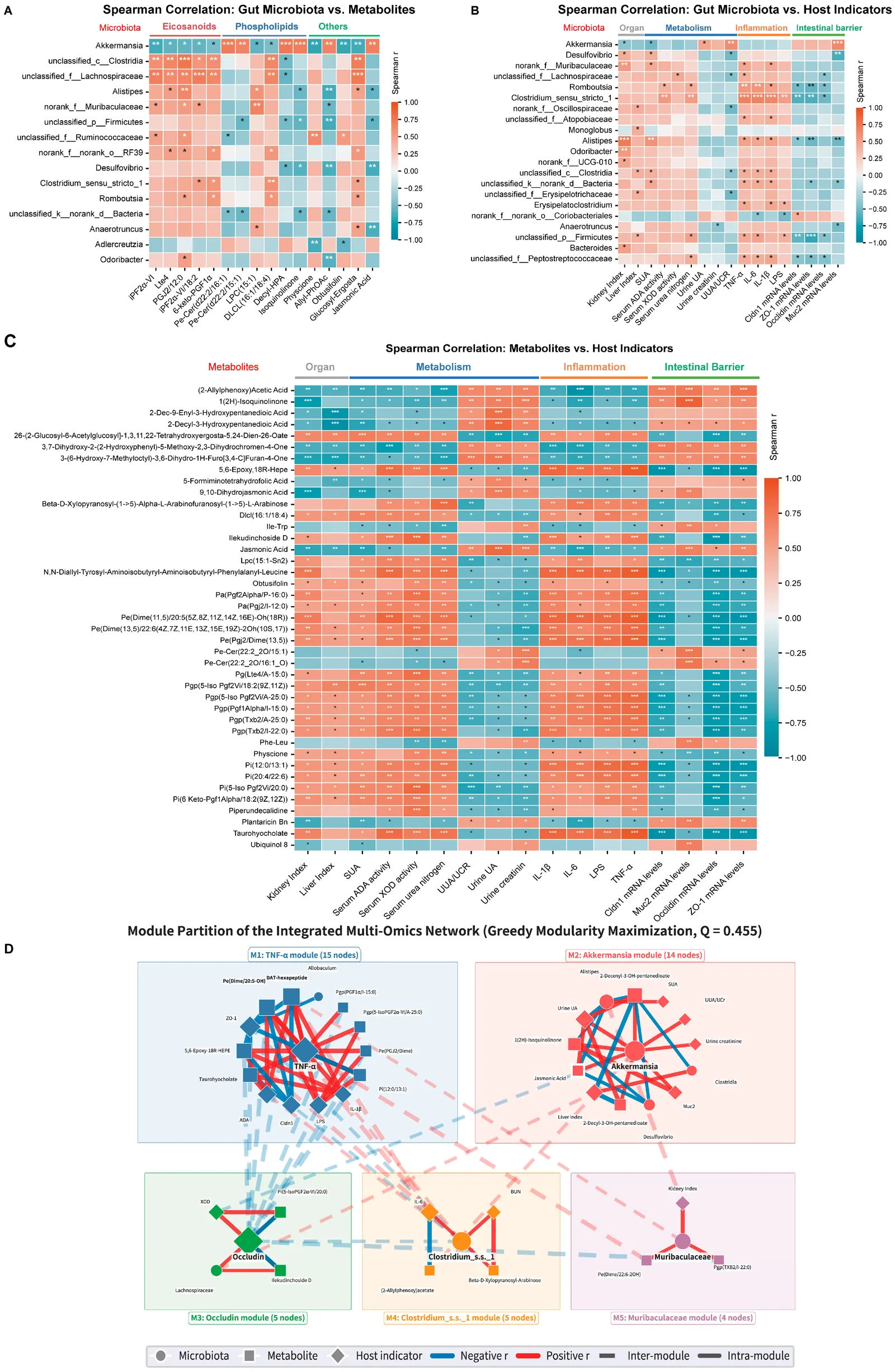
Multi-omics Spearman correlation analysis of gut microbiota, differential metabolites, and host indices in hyperuricemic mice. (**A**) Heatmap of Spearman correlations between dominant gut genera and fecal differential metabolites; (**B**) heatmap of Spearman correlations between gut genera and host biochemical indices; (**C**) heatmap of Spearman correlations between core differential metabolites and host biochemical indices; (**D**) module partition of the “microbiota–metabolite–host” multi-omics integration network by greedy modularity maximization (modularity Q = 0.455): M1, TNF-α module (15 nodes); M2, *Akkermansia* module (14 nodes); M3, *Occludin* module (5 nodes); M4, *Clostridium_sensu_stricto_1* module (5 nodes); M5, Muribaculaceae module (4 nodes). Node size is proportional to degree centrality; red and blue edges indicate positive and negative correlations, respectively. Significance: * *p* < 0.05, ** *p* < 0.01, *** *p* < 0.001.

Spearman correlation between microbial taxa and metabolites (Figure 5A) showed that *Clostridium_sensu_stricto_1* and *Alistipes*, which were enriched in the HUA group, correlated significantly and positively with arachidonic-acid-related metabolites, whereas *Akkermansia*, enriched in the CON and RES groups, correlated negatively with them. The associations between microbial genera and host indices are shown in Figure 5B. In the metabolite–host heatmap (Figure 5C), 10,11-dihydro-20-trihydroxy-leukotriene B4, the major metabolite of leukotriene B4 (LTB4), was strongly and negatively correlated with TNF-α (r = −0.884), whereas the peptide metabolite Leu-Ala-Thr-Lys was strongly and positively correlated with TNF-α (r = 0.913).

Integrating the microbiota, metabolites, and host phenotypes yielded a multi-omics association network (Figure 5D) comprising 42 nodes (9 microbial taxa, 17 differential metabolites, and 16 host phenotypic indices) and 46 significant edges (|ρ| ≥ 0.47, Benjamini–Hochberg-adjusted *p* < 0.05). Module detection partitioned the network into five functional modules (modularity Q = 0.455): M1 (TNF-α module, 15 nodes) centered on the pro-inflammatory cytokine TNF-α and its associated lipid mediators and barrier genes; M2 (*Akkermansia* module, 14 nodes) centered on *Akkermansia* and its associated purine metabolites (SUA, urine UA, UUA/Ucr) and microbial partners (*Alistipes*, Desulfovibrio, Clostridia); M3 (Occludin module, 5 nodes) centered on the tight junction protein Occludin and its associated microbes (*Lachnospiraceae*) and metabolites; M4 (*Clostridium_sensu_stricto_1* module, 5 nodes) centered on *Clostridium_sensu_stricto_1* and inflammatory markers (IL-6, BUN); and M5 (*Muribaculaceae* module, 4 nodes) centered on *Muribaculaceae* and renal injury indices (kidney index).

## 4. Discussion

### 4.1. Resveratrol Ameliorates Hyperuricemia Through Dual Inhibition of Urate Production and Restoration of Renal Function

This study systematically confirmed the multi-dimensional regulation of uric acid homeostasis by Res in HUA mice. Unlike benzbromarone, which lowers uric acid mainly by blocking tubular urate reabsorption, Res significantly reduced SUA while also inhibiting serum XOD and ADA activities, suggesting that its mechanism involves both suppression of urate production and protective restoration of renal filtration function. Previous studies have shown that Res lowers uric acid by directly inhibiting XOD activity and modulating the expression of renal urate transporters [34,35], and more recent work combining molecular docking with enzyme kinetics confirmed that Res acts directly on XOD in a mixed inhibition mode, giving it dual potential in suppressing urate production and inflammation [44]. Building on these findings, our results further indicate that the in vivo effect of Res is not achieved solely through direct hepatic XOD inhibition but through a systematic remodeling of the gut microbiota–metabolism axis, offering a new mechanistic interpretation of Res as a dietary intervention factor for HUA.

We would like to clarify that the positive control drug used in this study was benzbromarone, not allopurinol. Benzbromarone is a uricosuric agent that primarily lowers serum uric acid by inhibiting renal tubular urate reabsorption via URAT1, thereby enhancing urinary urate excretion without directly affecting hepatic XOD activity or intestinal urate handling. In contrast, Res exerts a broader, multi-compartment effect: it not only suppresses serum XOD activity but also enhances epithelial barrier integrity and may upregulate intestinal urate transporters (ABCG2, GLUT9), and remodels gut microbial structure and metabolic function. This distinction highlights the potential of Res as a comprehensive modulator of urate homeostasis through both systemic and intestinal mechanisms, rather than a single-target pharmaceutical approach.

We acknowledge that the mechanisms underlying Res-induced enhancement of intestinal urate excretion involve two distinct processes: (1) strengthening of epithelial tight junctions (ZO-1, Occludin, Claudin-1), which reduces paracellular permeability and limits back-diffusion of urate from intestinal lumen to blood, and (2) potential upregulation of apical and basolateral urate transporters (such as ABCG2 and GLUT9), which facilitate active transcellular secretion of urate into the intestinal lumen. While our data demonstrate improved barrier integrity, intestinal urate excretion was not directly measured in this study; the relative contribution of paracellular sealing versus transcellular transport to the overall urate-lowering effect therefore remains to be elucidated through targeted studies of transporter expression, localization, and functional activity, together with direct quantification of fecal and transepithelial urate excretion.

### 4.2. Modulation of Gut Microbial Composition by Resveratrol

Our 16S rDNA sequencing revealed a dysbiotic profile in HUA mice characterized by abnormally inflated richness, enrichment of Firmicutes- and Desulfobacterota-related lineages, and depletion of beneficial genera such as *Akkermansia*, which is broadly consistent with previous studies of HUA and gout patients [23,45,46]. After Res intervention, the overall community structure shifted toward the normal control, with the characteristic enrichment of *Akkermansia* being the most prominent feature: the magnitude of its restoration, together with the broader adjustment of phylum-level structures, exceeded that achieved by benzbromarone. As a mucin-layer-colonizing bacterium, reduced *Akkermansia* abundance is closely associated with barrier dysfunction and metabolic inflammation [47,48]. In our study, the Res-induced rebound of *Akkermansia* was accompanied by up-regulated expression of colonic tight junction proteins (Occludin, ZO-1, Claudin-1, and Muc2) and reduced serum LPS, suggesting that Res may repair the mucosal barrier by promoting *Akkermansia* colonization. Zhang et al. showed that both live and pasteurized *A. muciniphila* improve HUA symptoms in mice by modulating the gut microbiota, repairing the intestinal barrier, and regulating uric acid metabolism [26]; more recent evidence indicates that heat-inactivated *A. muciniphila* (a postbiotic form) still exerts anti-HUA effects by suppressing the TLR4/NF-κB/NLRP3 inflammatory cascade [27], an effect replicated in an avian model [28], implying that the benefits of *Akkermansia* may not depend entirely on live colonization and that bacterial components and their metabolic output also hold intervention potential. It is worth noting that benzbromarone also significantly enriched *Akkermansia*, but its microbial effect was largely confined to the single phylum Verrucomicrobiota; this contrast suggests that urate lowering alone creates conditions favorable for *Akkermansia* colonization, whereas Res exerts broader positive regulation of community structure, possibly related to its direct modulation of local intestinal inflammation, oxidative stress, and mucosal barrier integrity.

Notably, PICRUSt2 functional prediction suggested that the microbial purine-metabolism gene families *XOD/XDH* and *UOX* were markedly up-regulated in the HUA group, by 45.8% and 172.1% relative to the CON group, suggesting potential hyperactivation of purine degradation pathways by the gut microbiota. The work of Kasahara et al. confirmed that intestinal bacterial metabolism contributes to host global purine homeostasis [49]: microbial purine-metabolizing enzyme systems participate in the degradation and transformation of luminal purines and thereby influence the host uric acid load. The abnormal hyperactivation of microbial purine degradation under HUA conditions may thus constitute an important microbiological basis for disturbed intestinal urate handling. After treatment with Res at 80 mg/kg, these elevations were predicted to be reduced by 29.4% and 85.9%, suggesting that Res may not only remodel microbial composition but also potentially restore purine-metabolic functional homeostasis, in agreement with recent advances regarding the involvement of the gut microbiota in purine metabolism and HUA pathogenesis [50,51].

### 4.3. Regulation of Fecal Metabolite Profiles and Eicosanoid Metabolism

Untargeted fecal metabolomics showed that Res markedly reshaped the fecal metabolic profile of HUA mice. Among the 41 differential metabolites shared by the CON vs. HUA and HUA vs. RES comparisons, eicosanoids and lipid mediators were the dominant functional category (29.3%), followed by phospholipids and sphingolipids (17.1%). KEGG pathway enrichment further confirmed arachidonic acid metabolism, linoleic acid metabolism, and α-linolenic acid metabolism as the main perturbed pathways. Arachidonic acid metabolism is central to inflammatory responses and lipid signaling, and its reprogramming is closely linked to the pathogenesis of HUA and gout [52,53]. Under HUA conditions, monosodium urate crystals can activate the NLRP3 inflammasome and trigger the arachidonic acid cascade with production of pro-inflammatory lipid mediators. We found that eicosanoid and lipid mediator metabolites were significantly disturbed in the feces of the HUA group and were restored by Res, suggesting that Res may alleviate HUA-associated inflammation by correcting the arachidonic acid metabolic disturbance.

### 4.4. Exploratory Microbiota–Metabolite–Host Network Analysis

Multi-omics network analysis suggested a system-level picture: a potential *Akkermansia*-centered protective module (M2) may antagonize the *Clostridium_sensu_stricto_1*-centered module (M4) and the TNF-α-centered inflammatory module (M1). The major LTB4 metabolite 10,11-dihydro-20-trihydroxy-leukotriene B4 correlated significantly and negatively with TNF-α, whereas the peptide metabolite Leu-Ala-Thr-Lys correlated positively with TNF-α, suggesting that specific microbial taxa and their metabolites may serve as core nodes potentially linking dysbiosis to the host inflammatory state. The exploratory identification of this “microbiota–eicosanoid metabolite–host inflammation” axis potentially integrates two previously separate observations—microbial changes and inflammatory improvement—into an interpretable hypothesis.

### 4.5. Comparative Analysis with Other Dietary Interventions and Therapeutic Implications

The two genera characteristically enriched in the HUA group warrant separate consideration. Members of *Alistipes* are increasingly implicated in inflammatory and metabolic phenotypes [54], yet the genus appears functionally heterogeneous: *Alistipes indistinctus* was recently reported to produce hippuric acid, which promotes intestinal urate excretion and alleviates hyperuricemia [55], indicating that genus-level enrichment may mask species- and metabolite-specific effects. Butyrate provides a complementary example of how microbial metabolic output, rather than any single taxon, links dysbiosis to host urate handling. As the principal short-chain fatty acid generated from dietary fiber fermentation, butyrate has been proposed as a central bridge between dietary intervention and hyperuricemia [56]: it maintains colonic regulatory T-cell homeostasis [57], promotes the resolution of neutrophilic inflammation in a gout model [58], and enhances intestinal uric acid excretion [59]. Viewed together with our exploratory network results—in which metabolites, not taxa alone, appear to form the strongest connections to host phenotypes—these studies suggest that the benefit of microbial remodeling in HUA may lie in the restoration of a protective metabolic milieu, of which Akkermansia enrichment is one prominent marker.

*Akkermansia*-centered remodeling may in fact be a shared denominator of dietary intervention in HUA. In a human proof-of-concept study, pasteurized *A. muciniphila* improved insulin sensitivity in overweight and obese volunteers [60], supporting the translational plausibility of targeting this genus and its postbiotic potential. Beyond single-strain supplementation, a growing body of work has positioned plant-derived compounds as microbiota-directed regulators of urate homeostasis, although their entry points differ: rosmarinic acid and punicalagin both ameliorate hyperuricemia by restoring renal and intestinal function [61,62], whereas berberine acts upstream through *Bacteroides fragilis*-derived succinic acid, which inhibits hepatic adenosine monophosphate deaminase 2 (AMPD2) and accelerates uric acid degradation [63]. Compared with these compounds, Res appears distinctive in simultaneously correcting hyperactivated microbial purine degradation and reprogramming eicosanoid lipid mediator metabolism, a dual regulation that our multi-omics network exploration suggested as the coupling of the purine-metabolism and inflammatory modules. Elucidating whether these convergent interventions act through a common microbial effector—or complementary ones—will be important for rationally designing combined dietary strategies against HUA.

The pioneering study of Zhou et al. (2024) first confirmed that Res relieves HUA symptoms by remodeling the gut microbiota [36], laying an important foundation for the microecological mechanism of Res. Building on that work, we integrated untargeted fecal metabolomics with multi-omics association analysis: on the one hand, metabolomics systematically resolved the overall metabolic output of the microbiota under HUA conditions and after Res intervention, identifying eicosanoid lipid mediators as candidate metabolites potentially linking microbial changes with host phenotypes; on the other hand, the three-layer “microbiota–metabolite–host phenotype” network exploration suggested potential associations among microbial structural changes, metabolite alterations, and host uric acid/inflammatory phenotypes. Moreover, by benchmarking against benzbromarone, a first-line clinical urate-lowering drug, we observed differences in the breadth of microbial regulation: Res remodeled the community structure more broadly, whereas the microbial effect of benzbromarone was largely confined to *Akkermansia* enrichment, providing a new experimental perspective on the microecological differences between urate-lowering strategies.

### 4.6. Limitations and Future Directions

Several limitations should be acknowledged. First, the HUA model was induced by potassium oxonate combined with fructose over a four-week period. While this approach has been widely adopted in HUA research [41,64] it primarily reflects a chemically induced subacute hyperuricemic state rather than the fully chronic metabolic derangements observed in human patients. Additionally, the four-week intervention was relatively short, so the long-term efficacy and safety of Res require further evaluation. Future studies should consider extending the induction period, prolonging the intervention duration, or employing uricase-deficient genetic models to better recapitulate the chronic progression of human HUA and its renal complications.

Second, the doses of Res used in this study (20, 40, and 80 mg/kg/day in mice) correspond to Human Equivalent Doses (HEDs) of approximately 1.6, 3.2, and 6.5 mg/kg/day, respectively [65]. While these doses are achievable through dietary supplementation and have been demonstrated as safe in previous clinical trials [66], the higher dose approaches pharmacologic levels that may exert off-target effects. Future dose–response studies in humans should carefully evaluate the therapeutic window and potential gastrointestinal tolerability.

Third, we did not include a Res-only group without HUA induction, which would have helped distinguish Res-specific effects on gut microbiota and intestinal barrier from HUA rescue effects. However, our previous pilot study showed that Res alone at 80 mg/kg did not significantly alter serum uric acid or body weight in normal mice, suggesting that the microbiota remodeling observed in this study is primarily a response to HUA correction rather than a direct effect of Res on normal physiology. Future studies should incorporate a Res-only arm to definitively resolve this question.

Fourth, intestinal urate excretion was not directly quantified in this study: although Res lowered serum uric acid while restoring the intestinal barrier, enhanced intestinal urate excretion remains an inference that requires direct measurement, particularly because fecal uric acid is influenced by microbial uricolysis and would represent a net balance between epithelial secretion and bacterial degradation. Furthermore, while we assessed renal function through histology and serum creatinine/BUN measurements, we did not evaluate the expression or activity of renal urate transporters (URAT1, GLUT9, OAT1/3), which are the primary determinants of renal urate handling. Future studies should employ more direct functional assays, such as ex vivo Ussing chamber flux or everted gut sac preparations using isotope-labeled uric acid, to precisely quantify transepithelial urate transport and to distinguish Res-mediated transcellular secretion from microbial degradation effects; renal transporter expression and activity should also be investigated to comprehensively distinguish between intestinal and renal mechanisms of action.

Fifth, although PICRUSt2 predicted alterations in microbial purine-metabolism pathways, these are computational predictions based on 16S rDNA sequence homology rather than direct measurements of gene expression or enzymatic activity. Future studies should employ metagenomic and metatranscriptomic sequencing to confirm the presence, abundance, and transcriptional activity of specific purine-degradation genes such as ucnA, thereby providing more definitive evidence for Res-induced remodeling of microbial purine metabolism.

Sixth, with only six animals per group, the sample size for the 16S rDNA sequencing and metabolomics analyses was modest, and the multi-omics network merged only 18 samples from the CON, HUA, and RES groups. Although differential abundance testing, metabolite screening, and Spearman correlation analysis were all corrected for multiple testing using the Benjamini–Hochberg false discovery rate (FDR) procedure, the limited sample size may still reduce statistical power and constrain the detection of low-abundance taxa and weak associations. More importantly, all microbiota–metabolite–host associations in this study rest on Spearman rank correlation and are therefore correlational rather than causal evidence; without antibiotic depletion or fecal microbiota transplantation arms, the necessity of microbial remodeling for the urate-lowering effect of Res remains to be confirmed through gnotobiotic colonization with single strains, antibiotic clearance, and fecal microbiota transplantation [67].

## 5. Conclusions

Using a mouse model of HUA induced by potassium oxonate combined with high-fructose intake, this study integrated 16S rDNA sequencing, PICRUSt2 functional prediction, untargeted fecal metabolomics, and multi-omics integration analysis to explore the mechanism by which Res ameliorates HUA through the “gut microbiota–metabolism” axis. While suppressing urate production, Res remodels gut microbial composition, restores microbial purine-metabolic homeostasis, reprograms arachidonic-acid-derived lipid mediator metabolism, and repairs the intestinal mucosal barrier. Its mode of action differs from the uricosuric mechanism of benzbromarone and presents a combined pattern of “urate-production suppression plus microbiota–metabolism axis regulation”, providing preclinical experimental evidence supporting further investigation of Res-based dietary interventions for HUA. However, given the poor oral bioavailability and rapid biotransformation of Res, further pharmacokinetic optimization and clinical validation are warranted before therapeutic application.

## Figures and Tables

**Table 1:** Primer sequences for RT-qPCR.

Name	Forward Primer (5′→3′)	Reverse Primer (5′→3′)
*GAPDH*	ACTCCACGACATACTCAGCA	CATCAACGACCCCTCATT
*ZO-1*	AGCGAAGCCACCTGAAGATA	GATGGCCAGCAGGAATATGT
*Occludin*	AAGAGTCGACAGTCCAATGG	TGAAGTCATCCACGGACAAG
*Muc2*	GAGGACAGGCCCATCTATGA	CAGATCCTCCAGGTGGGTAG
*Claudin-1*	ACCCCATGACCCCTATCAAT	CGTGGTGTTGGGTAAGAGGT

## Data Availability

The 16S rRNA sequencing data presented in this study are openly available in the NCBI Sequence Read Archive (SRA) under BioProject accession number PRJNA1531718 (https://www.ncbi.nlm.nih.gov/sra?term=PRJNA1531718, accessed on 19 September 2026). The metabolomics data supporting the conclusions of this article are available from the corresponding authors upon reasonable request.
